# Long-segment fixation VS short-segment fixation combined with kyphoplasty for osteoporotic thoracolumbar burst fracture

**DOI:** 10.1186/s12891-022-05109-y

**Published:** 2022-02-17

**Authors:** Oujie Lai, Xinliang Zhang, Yong Hu, Xiaoyang Sun, Binke Zhu, Weixin Dong, Zhenshan Yuan

**Affiliations:** 1 Department of Spine Surgery, Ningbo No.6 Hospital, Ningbo, Zhejiang People’s Republic of China; 2 Department of Spine Surgery, Xi’an Honghui Hospital, Xi’an Jiaotong University, Xi’an, Shaanxi People’s Republic of China

**Keywords:** Thoracolumbar burst fracture, Osteoporosis, Short-segment, Long-segment, Kyphoplasy

## Abstract

**Background:**

To retrospectively compare clinical and radiological results of long-segment fixation (LF) and six-screw short-segment fixation combined with kyphoplasty (SSFK) for osteoporotic thoracolumbar burst fracture (OTBF).

**Methods:**

Forty patients affected by OTBF with mean age of 61.85 years were included in this study. The mean follow-up period was 13.63 months. Twenty-four patients were treated by SSFK, and 16 patients were treated by LF. Clinical outcomes, radiological parameters and complications were assessed and compared.

**Results:**

The mean operative time and blood loss were 89.71 ± 7.62 min and 143.75 ± 42.51 ml for SSFK group, respectively; 111.69 ± 12.25 min (*P* < 0.01) and 259.38 ± 49.05 ml (*P* < 0.01) for LF group, respectively. The two groups were similar in terms of preoperative radiological and clinical results. Compared with preoperative values, both groups achieved significant improvement in terms of VAS, ODI, Cobb angle and anterior vertebral body height (AVH) ratio at final follow-up. However, during the follow-up period, significant loss of Cobb angle and AVH ratio were observed for both groups. Five cases (20.83%) of asymptomatic cement leakage were observed in SSFK group. One case of implant failure and two cases of adjacent or non-adjacent vertebral fractures were observed in LF group.

**Conclusions:**

Both SSFK and LF are safe and effective for treatment of OTBF. Comparatively, SSFK is less invasive and can preserve more motion segments, which may be a more valuable surgical option in some elderly patients. A high-quality randomized controlled study is required to confirm our finding in the future.

## Background

Thoracolumbar burst fracture in elderly osteoporotic patients is one of the most challenging issues in spinal traumatology, who generally have more accompanying diseases including cardiopulmonary problems, cerebrovascular disease, diabetes and hypertension [1]. The optimal surgical strategy remains debated when surgical treatment is indicated. Anterior approach or combined surgery can directly reconstruct anterior column, decompress neurological structure and provide superior biomechanical stability. However, these surgeries are fraught with complication due to debilitated state of elderly patients [2, 3]. When posterior approach is performed, controversy exists regarding the use of fixation by short or long-segment pedicle screws [4]. Posterior six-screw short-segment pedicle instrumentation (include the fractured vertebra) is recommended by some surgeons for the treatment of thoracolumbar burst fracture, in order to reduce surgical invasiveness, enhance biomechanical stability and minimize the number of fixation segments [5]. However, for osteoporotic thoracolumbar burst fracture (OTBF), short-segment fixation is reported to show less favorable results in terms of kyphotic correction, correction maintenance and implant failure compared with those of long-segment fixation (LF) due to poor anterior column support and lower mechanical stiffness, even six-screw short-segment technique have been used [6, 7].

Percutaneous kyphoplasty (PKP) is a minimal invasive and reliable treatment for osteoporotic compression fracture, which can reduce pain, restore vertebral heigh and augment anterior column [8, 9]. Due to the encouraged results, some authors even expanded this technique to OTBF as a stand-alone intervention for eliminating the need of major operation [10]. Although stand-alone cement augmentation could provide some degree of support for the anterior column, persistent traumatic instability of the affected vertebra was still reported [11, 12]. Hence, stand-alone cement augmentation for burst vertebral fracture should not be advocated.

A technique of combining posterior six-screw short-segment fixation with kyphoplasty (SSFK) had been reported for the treatment of OTBF, which theoretically combined the advantages of two relatively less invasive procedures, and enhanced postoperative biomechanical stability [13]. However, there are no clinical studies available comparing the effect of SSFK and LF on OTBF, and it’s not clear whether SSFK could achieved similar results as the LF. In this study, we aimed to evaluate and compare the clinical and radiological results in patients with OTBF treated by LF or SSFK.

## Materials and methods

In this study, we retrospectively reviewed the results of thoracolumbar burst fracture in consecutive 40 surgically treated osteoporotic patients without neurological deficit at our institution from January 2016 to January 2019. Surgical indications included more than 50% loss of anterior vertebral body height (AVH), regional kyphotic deformity more than 20 ° or significant posterior element lesion. The inclusion criteria included (1) single-level AO A3 or A4 type burst fracture between T11-L2 [2, 14] osteoporosis: mean T score by BMD (bone mineral density) < − 2.5; (3) no neurological deficits; (4) less than 2 weeks from the time of injury to surgery. The exclusion criteria were as follow: (1) two or more levels thoracolumbar burst fractures; (2) pathological fracture; (3) inflammatory diseases (ankylosing spondylitis, rheumatoid arthritis or active infection); (4) a history of major thoracolumbar spinal surgery. The study protocol was approved by the Institutional Review Board of the authors’ institute.

There were 24 women and 16 men with an average age of 61.85 ± 7.36 years (range 44–76 years) in our study. The level of burst fracture was T11 in 4 patients, T12 in 8 patients, L1 in 20 patients and L2 in 8 patients. Fifteen of these injuries were motor accident, twelve were ground fall, five were fall from a height, seven were crush injury and one was spontaneously.

**Fig. 1:**
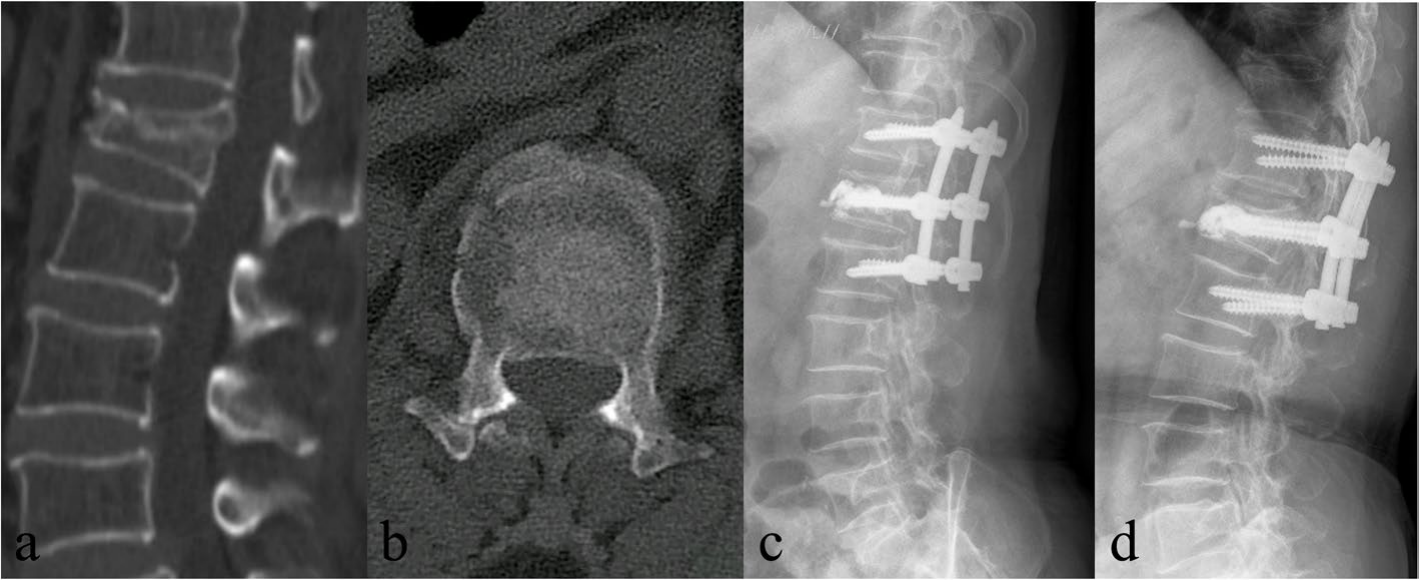
**a-b** Preoperative Sagittal and axial CT images of a 71-year-old female showing L1 burst fracture; **c** Immediate postoperative lateral plain radiograph showing AVH and kyphotic deformity were corrected after SSKF treatment; **d** Plain radiograph at the final follow-up

**Fig. 2:**
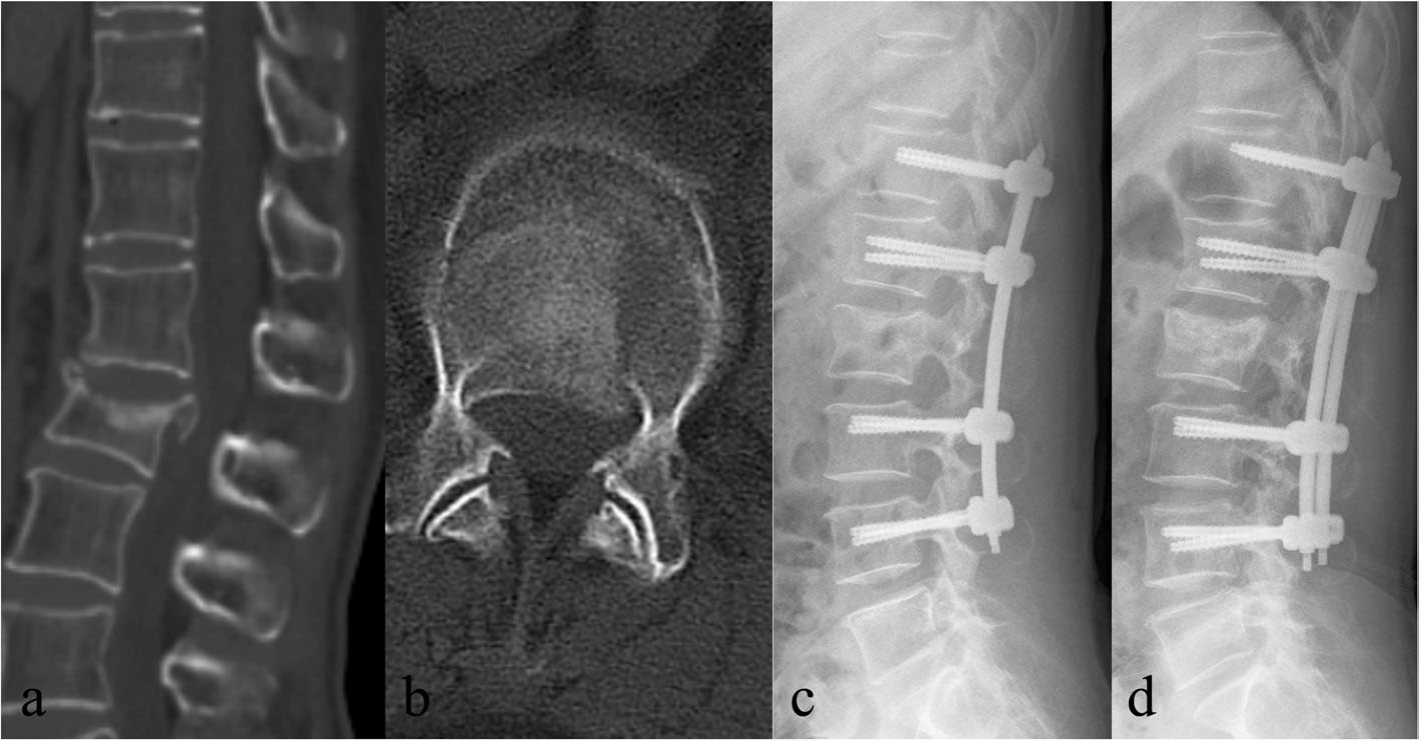
**a-b** Preoperative Sagittal and axial CT images of a 62-year-old female showing L2 burst fracture; **c** Immediate postoperative lateral plain radiograph showing AVH and kyphotic deformity were corrected after LF treatment; **d** Plain radiograph at the final follow-up

The 40 patients were divided into two groups by surgical techniques. Twenty-four patients were treated by posterior six-screw short-segment fixation combined with fractured vertebra kyphoplasty (SSFK group) (Fig. 1). The left sixteen patients were treated by posterior long-segment fixation (LF group) (Fig. 2). The differences between SSFK and LF were explained to all the patients before surgery, and they selected the surgical method according to their preference.

### Surgical technique

All patients were positioned in prone hyperextended position under general anesthesia with pillows under the upper chest and pelvis to facilitate the postural reduction of the fractured vertebral body. A posterior midline incision was adopted, and pedicle screw insertion points were exposed through the paraspinal sacrospinalis muscle-splitting approach duo to intact neurological status and no laminectomy performed [15]. For SSFK group, bilateral pedicle screws one level above and below the affected vertebra and unilateral pedicle screw of the affected vertebrae were placed into the vertebral body using free-hand technique. Bilateral contoured distraction rods were used to restore the vertebral body height and correct the regional kyphosis. Under fluoroscopic guidance, trocar and cannula system were sequentially driven into central portion of the fractured vertebral body through the unscrewed transpedicular trajectory to establish working channels [7, 16]. The balloon was inserted into the vertebral body, and then inflated slowly to restore the vertebral body height further. Doughy bone cement was slowly injected into the fractured vertebrae under fluoroscopic control. The rod at the unscrewed side of the fractured vertebrae was removed, and pedicle screw was placed through the cement augmentation trajectory, and then the rod was reconnected and tightened. For LF group, pedicle screws were placed into the vertebrae two levels above and below the affected vertebra through the paraspinal sacrospinalis muscle-splitting approach. The affected vertebral body height was restored by means of bilateral rods contouring and cantilever reduction of the rods into the screw heads. All the patients were encouraged to mobilize as soon as feasible after surgery, and thoracolumbosacral orthosis was used for 3 months after surgery. Anti-osteoporosis treatments were performed for both groups after surgery.

### Clinical and radiological evaluations

The preoperative, postoperative and final follow-up clinical and radiological assessments were performed for all the patients. The operative time, blood loss and complications were recorded by reviewing the medical records. The visual analog scale (VAS) ranging from 0 (no pain) to 10 (maximal pain) was used to evaluate pain severity. Oswestry Disability Index (ODI) was used to evaluate functional outcome.

The kyphotic deformity was evaluated preoperatively, postoperatively and at final follow-up using the Cobb angle. The Cobb angle was measured between the superior endplate of the upper vertebra and inferior endplate of the lower vertebrae at the fracture site. The correction of Cobb angle after surgery and correction loss during the follow-up period were calculated accordingly. The AVH ratio was calculated by the AVH of the injured vertebra to the mean anterior height of the adjacent above and below intact vertebrae. Bone cement leakage was defined as any cement which was out of the confines of the vertebral body, which was evaluated through immediately postoperative computed tomography.

### Statistical analysis

SPSS software (version 20.0; SPSS Inc., Chicago, IL, USA) was used for statistical analysis. Continuous variables in this study were presented as means ± standard deviation. Intragroup comparisons were made using the Wilcoxon signed-rank test, and intergroup comparisons were made using the Mann-Whitney U test. A result was statistically significant with *p*-value < 0.05.

## Results

### Clinical outcomes

The follow-up duration of the 40 patients was 13.63 ± 3.53 months. The two groups were similar in terms of age, gender, BMD, fracture level and follow-up duration (Table 1). The mean operative time was 89.71 ± 7.62 (78–110) min and 111.69 ± 12.25 (95–136) min for SSFK group and LF group, respectively (*P* < 0.01); the mean blood loss was143.75 ± 42.51 (100–200) ml and 259.38 ± 49.05 (200–300) ml, respectively (*P* < 0.01).

**Table 1:** Baseline demographic and clinical characteristics of SSFK Group and LF Group

	SSFK group	LF group	*P* value
Number of patients	24	16	–
Age	63.42 ± 6.93	59.5 ± 7.36	0.126
Gender (F/M)	10/14	6/10	0.838
BMD	− 3.21 ± 0.59	−3.42 ± 0.57	0.436
Fracture level	T11(2) T12(4) L1(13) L2(5)	T11(2) T12(4) L1(7) L2(3)	0.557
Follow-up duration (month)	13.17 ± 3.89	14.31 ± 2.89	0.292

*F* Female, *M* Male

Both preoperative VAS and ODI were similar between the two groups (Table 2). For both groups, VAS and ODI decreased significantly over time after surgery. No significant differences in terms of VAS and ODI were found between the two groups at the final follow-up.

**Table 2:** The changes of VAS and ODI of the two groups before and after surgery

VAS				ODI (%)		
	SSFK Group	LF Group	P value	SSFK Group	LF Group	P value
Preoperative	8.71 ± 0.89	8.43 ± 0.79	*P* = 0.41	81.32 ±8.74	82.57 ± 9.45	*P* = 0.55
Final follow-up	2.21 ± 1.00	2.69 ± 1.10	*P* = 0.22	25.35 ± 5.31	24.68 ± 6.46	*P* = 0.43
P value	*P* < 0.01	*P* < 0.01		*P* < 0.01	*P* < 0.01	

### Radiological outcomes

There was no significant difference between the two groups for preoperative, immediately postoperative and final Cobb angle (Table 3). Both groups achieved significant improvements of kyphotic deformity after surgery (*P* < 0.01 for both groups), and the kyphotic correction was 12.59 °± 4.82 ° in SSFK group and 12.69 °± 5.67 ° in LF group (*P* = 0.967), respectively. During follow-up period, the loss of correction was 6.04 °± 4.30 ° in SSFK group and 7.53 °± 4.82 ° in LF group (*p* = 0.576), respectively, which resulted significant difference between immediately postoperative and final Cobb angle (*P* < 0.01 for both groups). However, final Cobb angle still showed significant improvement compared with preoperative values for both groups (*P* < 0.01 in SSFK group and *P* = 0.013 in LF group).

**Table 3:** The Cobb angle before and after surgery

	Preoperative (°)	Postoperative (°)	Final follow-up (°)
SSFK Group	11.45 ± 6.45	−1.14 ± 6.43	4.90 ± 8.00
LF Group	14.57 ±6.33	1.88 ±8.07	9.41 ± 8.26
P value	0.070	0.345	0.066

In SSFK group, the AVH ratio was 64.81% ± 9.33% preoperatively, 90.71% ± 4.13% postoperatively and 85.71% ± 4.39% at final follow-up, respectively. Compared with SSFK group, the AVH ratio of LF group was 59.62% ± 10.96% preoperatively (*P* = 0.183), 91.78% ± 3.54% postoperatively (*P* = 0.503) and 83.30% ± 13.73% at final follow-up (*P* = 0.859), respectively. Both groups achieved significant AVH ratio restoration after surgery (*P* < 0.01 for both groups). There was significant correction loss for both group (*P* < 0.01 for both groups) during the follow-up period, however, the final AVH ratio still showed significant improvement for both groups (*P* < 0.01for both groups) compared with the preoperative values. Between the two groups, there was no significant difference regarding the AVH restoration and loss (Table 4).

**Table 4:** The AVH ratio correction and loss

	SSFK group	LF group	P value
AVH correction (%)	25.89 ± 9.37	32.16 ± 10.81	0.070
AVH loss (%)	5.00 ±2.36	7.23 ±7.40	0.267

### Complications

No postoperative infection or neurological injury occurred in this study. At the final visit, screw loosening was observed in two patients in SSFK group (8.33%) and two patients in LF group (12.5%), respectively. Due to instrumentation failure, one patient in LF group had significant postoperative re-collapse of the affected vertebra. However, revision surgery was still under the patient’s consideration, as the low back pain was not severe. Five cases (20.83%) of asymptomatic cement leakage were observed in SSFK group. Two cases of adjacent or non-adjacent vertebral fracture occurred in LF group (12.5%), and one was treated by PKP and the other was treated conservatively.

## Discussion

The aim of this retrospective study was primary to evaluate the clinical and radiological outcomes of SSFK for OTBF, and to compare the outcomes with those of LF. Our results showed that both SSFK and LF could effectively reduce pain, improve function and correct kyphotic deformity for OTBF. However, compared with LF, SSFK had the advantage of reducing operative time and blood loss.

Although more and more OTBF happen nowadays due to an increasing aged population, the optimal surgical treatment for this type of fracture in aged patients remains a matter of discussion [17]. The goal of surgical treatment for OTBF patient without neurological deficit is to correct kyphotic deformity, provide sufficient biomechanical stability for early mobilization, while reduce surgical invasiveness and related complications. However, some challenges should be taken into consideration when choosing the surgical procedure, including poor bone quality, old age, medical comorbidities and possible perioperative morbidities.

Compared with anterior or combined approaches, posterior approach does not pose risks to chest or abdominal organs, and it is correlated with less surgical invasiveness and lower complication [18]. Further, most spine surgeons are more familiar with posterior approach. In biomechanically, LF with two or more levels above and below the fracture is a better choice for thoracolumbar burst fracture, which can provide greater mechanical stiffness and reduce likelihood of segmental collapse and implant failure [4, 19]. However, except disruption of spinal motion segments, LF is correlated with more severe surgical invasiveness. In our study, compared with SSFK group, the operative time and blood loss were significant higher in the LF group. In addition, LF construct may correlated with adjacent or non-adjacent vertebral fracture. Short-segment pedicle fixation has some advantages including less surgical invasiveness, preserving motion segment and reducing adjacent segment stress. While, unacceptable increasing instrumentation failure and kyphotic correction loss were reported after traditional four-screw short-segment (one level above and below the fracture) fixation for thoracolumbar burst fracture [20]. A finite element analysis for simulating burst fracture demonstrated four-screw short-segment posterior fixation permitted a greater range of motion (ROM) in flexion compared with intact condition, and the stability could be enhanced with the increased number of instrumented levels [21].

Six-screw pedicular fixation involving the placement of two pedicle screws at the fractured vertebra has been proposed to improve postoperative stability, meanwhile, retain the own advantages of short-segment fixation. Baaj et al. [5] reported adding bilateral index-level screws to short-segment constructs could improve stability by 25%, especially for flexion and lateral bending restriction, although the stability remained less than that provided by long-segment construct with or without index-level pedicle screws. Dobran et al. [22] even reported that six-screw short-segment construct for unstable thoracolumbar fracture resulted in a kyphosis correction and in a maintenance of sagittal alignment as a long-segment construct. However, the included patients in their study were relatively young, and the results were not based on the osteoporotic population. Schulze et al. [23] reported significant migration of pedicle screws following fixation of osteoporotic vertebrae placed under flexion/extension cyclic loading, and the anchoring effect holding the screw in place was decreased in osteoporotic cases. Although intermediate pedicle screws were used in the fractured vertebra, significant kyphotic correction loss and mechanical failure were observed in cases with osteoporotic thoracolumbar fracture [4]. In addition, the intravertebral area of osteoporotic burst fracture enlarged by positional or instrumental reduction is nearly empty, and such insufficient anterior column support may not endure vertical physiological strength with stand-alone posterior pedicle construct, even LF construct was used [24, 25].

The reconstruction of weight-bearing anterior column could reduce posterior instrumentation strain and sequentially reduce instrumentation failure and kyphotic correction loss, especially for osteoporotic thoracolumbar burst fracture. With the successful application of PKP in the treatment of osteoporotic vertebral compressive fracture, some authors even applied it to burst fracture [26]. Biomechanical study showed cement augmentation could supported the anterior column especially in flexion, however, did not reduced ROM in extension [11]. Hence, kyphoplasty of the affected vertebra combined with posterior instrumentation was proposed by some authors in order to achieve circumferential fixation through a single posterior approach [12]. By using this method, better VAS score reduction, ODI improvement and kyphotic correction could be achieved compared with simple PKP [12]. In a finite element analysis, Liao et al. [13] reported short-segment fixation combined with intermediate screws and anterior column cement augmentation provided the strongest stability among different types of posterior short-segment fixation. In our study, SSFK construct could provide sufficient stability, and no instrumentation failure or revision surgery was occurred in any patient.

Cement leakage is one of risks during vertebral augmentation for burst fracture due to the rupture of the posterior vertebral body wall and spinal canal occupancy [26]. Wang et al. [27] reported the cement leakage rate was 14.3% after using PKP for very severe osteoporotic vertebral compression fracture (vertebral body collapse to less than one third of their original height) with spinal canal compromise. However, in a study of comparing the results between osteoporotic burst and compression fractures with PKP, Li et al. [28] reported cement leakage rate was 47.8 and 36.6%, respectively, and all the leakages were minor and without neurological deficit. They conclude that osteoporotic burst fracture with asymptomatic spinal canal compromise was not a contraindication for PKP. We found the osteoporotic burst fractured vertebra exhibited an “eggshell” like change after postural and instrumental reduction, which created a relatively safe cavity for padding of thick and doughy bone cement. Further, due to posterior fixation supplementation, it is not necessary to inject bone cement into the fractured vertebral body as much as possible. In our study, the cement leakage rate (20.83%) was relatively low, and there was no case of leakage causing neurological deficit and organ compression. Hence, bone cement augmentation used in our study was a safe measure.

There are several limitations to the current study. Retrospective analysis reduced the evidence level. A small sample size was another weak point, limiting its statistical power. The follow-up duration in this study was relatively short. An extended observation period is beneficial for better evaluation of clinical efficacy, correction loss and instrumentation failure. Hence, a high-quality randomized controlled study of with larger sample size and longer follow-up duration is required to confirm our finding in the future.

## Conclusions

In conclusion, the results of this study show that SSFK and LF techniques have similar clinical and radiological results for the treatment of OTBF, and both are safe and effective. Comparatively, SSKF can achieve circumferential fixation for OTBF with less surgical invasiveness and fewer motion segments, which may be a more valuable surgical option in some elderly patients.

## Data Availability

The datasets used and/or analyzed during the current study are available from the corresponding author on reasonable request.
